# Better in theory than in practise? Challenges when applying the luck egalitarian ethos in health care policy

**DOI:** 10.1007/s11019-020-09962-3

**Published:** 2020-06-21

**Authors:** Joar Björk, Gert Helgesson, Niklas Juth

**Affiliations:** 1 Stockholm Centre for Healthcare Ethics (CHE), LIME, Karolinska Institutet, Tomtebodavägen 18 A, 171 77 Stockholm, Sweden; 2 Department of Research and Development, Region Kronoberg, PO Box 1223, 351 12 Växjö, Sweden

**Keywords:** Responsibility, Priority setting, Luck egalitarianism, Clinical ethics, Smoking

## Abstract

Luck egalitarianism, a theory of distributive justice, holds that inequalities which arise due to individuals’ imprudent choices must not, as a matter of justice, be neutralized. This article deals with the possible application of luck egalitarianism to the area of health care. It seeks to investigate whether the ethos of luck egalitarianism can be operationalized to the point of informing health care policy without straying from its own ideals. In the transition from theory to practise, luck egalitarianism encounters several difficulties. We argue that the charge of moral arbitrariness can, at least in part, be countered by our provided definition of “imprudent actions” in the health area. We discuss the choice for luck egalitarianism in health care between *ex ante* and *ex post* policy approaches, and show how both approaches are flawed by luck egalitarianism’s own standards. We also examine the problem of threshold setting when luck egalitarianism is set to practise in health care. We argue that wherever policy thresholds are set, luck egalitarianism in health care risks pampering the imprudent, abandoning the prudent or, at worst, both. Furthermore, we claim that moves to mitigate these risks in turn diminish the normative importance of the ethos of luck egalitarianism to policy. All in all, our conclusion is that luck egalitarianism cannot be consistently applied as a convincing and relevant normative principle in health care policy.

## Introduction

Luck egalitarianism (LE) is a theory of distributive justice, first elaborated during the 1980s (Knight and Stemplowska 2011). While it shares the strong emphasis on equality common to all brands of egalitarianism, LE provides a justification for pockets of inequality—if the inequality has arisen in a way that meets certain desiderata. One may thus speak of LE as a kind of *tempered egalitarianism*. The desiderata in question concern issues of *choice and risk taking*, and so (voluntary) choice has a special place in LE theory as the normative force distinguishing LE from outcome egalitarianism. Whereas outcome egalitarianism holds that inequality between individuals should be neutralized (*ceteris paribus*), LE holds that this rule applies except when the inequality has arisen due to the individuals’ *voluntary choices*. The term “luck” in “luck egalitarianism” derives from a pivotal 1981 article by Ronald Dworkin, which discusses the policy implications of what Dworkin calls “brute luck” and “option luck”, respectively (Dworkin 1981). In common parlance, “option luck” is translatable to *the outcomes of voluntary choices*.

If it is accepted, luck egalitarianism may be of great relevance to health care ethics, especially in matters of priority setting^1^. One application of LE to the health care arena, here labelled “luck egalitarianism in health care” (LEHC), argues that there is no injustice in situations where someone has worse health than another, if this inequality is due to the person’s voluntary choices in the health area (Bognar and Hirose 2014). Consequently, a proponent of LEHC would hold^2^ that there is no justice-based call to remedy such health inequality, and giving health care to those whose health is worse as a result of their own past choices should not be prioritized. As a clinical example, LEHC typically holds that a smoking lung cancer patient should be down-prioritized for treatment in comparison with a non-smoking patient (who presumably did not invite the disease by risk taking behaviour). Exactly how such down-prioritization is achieved—by giving the smoking lung cancer patient less treatment than the non-smoking patient, inferior treatment compared to that of the non-smoking patient, or treatment after the non-smoking patient – are practical questions that will not be dealt with here.

It should be noted that by most accounts the ethos of LEHC applies only *in situations of scarcity* (Segall 2009; Duus-Otterström 2012). That is, LEHC would down-prioritize the smoking lung cancer patient above only as long as *not* doing so would make the non-smoking lung cancer patient worse off. This is because the justification of down-prioritization in LEHC derives from a consideration of the harm or opportunity cost to third parties that arise as a result of one person’s imprudent^3^ health behaviour. (For a fuller background of the notion of opportunity cost, see Drummond et al. 2015). Here, the relevant third parties are prudent *patients*, prudent *tax payers*, or *both*. Two different kinds of opportunity costs are relevant in this context. The first is the cost in terms of lost health (or health care) that would befall the non-smoker if the two patients described above were treated similarly in a situation of scarcity. The second is the cost in financial terms that would befall prudent tax payers or insurance premium payers if they have to co-finance the extra health care needs generated by others’ imprudent actions^4^.

The concern to protect the prudent third party from any costs generated by others’ imprudent actions is important to differentiate LEHC from theories of desert (Segall 2009). The matter is of some importance, as some policy choices made by ‘desertists’ coincide with those suggested by the LEHC ethos. Yet the theories differ regarding their underlying motive. The best way to set the theories apart is to remember that LEHC is an offspring of egalitarianism, whereas desertism is not. Although desertism may occasionally function to equalize inequalities, it does not care about equality as such. Thus, in situations where there is no opportunity cost LEHC would strive to neutralize all health inequalities, whereas desertism would not necessarily do so^5^.

This article will proceed to examine the merits of LEHC critically. In so doing we will accept, for the sake of argument, what we see as the core ethos of LEHC: that health care policy should reflect individuals’ responsibility for their own health. What we seek to investigate, more specifically, is whether this ethos can be operationalized to the point of informing real world policy without straying from its own ideal. First, we will claim that a recurrent critique of LEHC—that it relies on *morally arbitrary* assumptions—can actually be met by drawing up a principled definition of imprudence, at least to some extent*.* In doing this, we clarify what critique LEHC can avoid, which brings us to the more fundamental problems with LEHC. Moving on, we will discuss the important choice of *ex post* versus *ex ante* policy approach in LEHC. We will claim that the *ex post* approach is plagued by epistemic difficulty and furthermore that it targets only a subset of the imprudent, without there being an ethical difference between this subset and the subset which is not targeted by the policy. This makes the *ex post* approach unsuitable as an expression of the LEHC ethos. However, the *ex ante* approach also targets only a subset of the imprudent, and furthermore it severs the link between imprudent action and outcome in a way that undermines the justificatory strength of the LEHC position. Thus, we propose that none of these approaches work to the benefit of LEHC, yet we see no third approach. We then turn to the question of policy threshold setting in LEHC, and propose that using a circumscribed version of LEHC risks “pampering the imprudent” and making LEHC nearly irrelevant. Conversely, using a more impactful LEHC version invites charges of abandoning negligent victims, unless LEHC is made part of a pluralist value theory. Coupling LEHC to other, overriding normative theory elements also risks robbing LEHC of much of its normative importance.

All in all, we believe that even despite its intuitive punch, LEHC is unconvincing as a theory of distributive justice in health care. This remains the case even if the theory is made as strong as possible.

Before undertaking this task, we should like to acknowledge that our discussion by no means exhausts the possible challenges to LEHC. For instance, several practical problems regarding the set-up of LEHC policy remain (see above). Nevertheless, if the arguments that we put forth are successful, they point to more fundamental problems, making the need of further fine-tuning of practical details less imperative.

## The charge of moral arbitrariness and the morally relevant dimensions in LEHC

In the *Nichomachian principles*, Aristotle states what has become known as “the principle of formal justice”. This principle emphasizes that two parties (individuals or groups of individuals) may be treated differently only if there is a morally relevant difference between them. Conversely, the parties should be treated equally if there are no morally relevant differences between them. The spirit of this principle has been invoked in the criticism of LEHC. Thus, previous authors have argued that it is impossible to discern between prudent and imprudent patients in a morally salient way (Wilkinson 1999; Wikler 2002), and that some of the groups claimed to be imprudent are in fact not, or not in the right way, imprudent (Walker 2010). Hence, a recurrent critique against LEHC is that it hinges on morally irrelevant differences between the (perceived) prudent and imprudent.

We believe, however, that this criticism need not be decisive against LEHC. Indeed, we will suggest a way to define *imprudence in health* that is both morally salient and suitable to the formulation of LEHC policy. Our core proposition is that imprudence, in the sense relevant to LE and LEHC, should be understood as *a morally unacceptable consumption of common resources*. By making reference to common resources, as those in public health care funds or pools of insurance capital, the notion of opportunity cost is accounted for. Thus, my behaviour is imprudent in the LEHC sense only if it increases my health care demand to a degree which infringes upon the satisfaction of the health care demands of another person, or if it increases the costs for those that pay for healthcare through taxation or insurance premiums.

While this proposition is necessary in order to avoid the objection that LEHC is morally arbitrary, it is not yet sufficient. To complete our model of imprudence, we propose four desiderata that we claim make the model robust against charges of harshness and moral arbitrariness. The suggested desiderata concern the *risk* of resource consumption, the *size* of this consumption, the *avoidability* of the action as well as the *insight*^6^ into all of those factors, so that:

An action (or set of actions) may be judged as imprudent if, and only if, it entails a high risk of a great size of consumption of common resources, could have been easily avoided by the agent, and the agent has high insight regarding the abovementioned risk, size of consumption and avoidability of the action.

As an illustration we may use a situation where I consider going rock climbing in a highly challenging area (= high risk/probability). Let us assume that I am under no other pressure to go rock climbing than that I am curious to do so (= high avoidability). Assume further that I am a poor rock climber, so that there is an increased risk of injury if I climb, and that if I were injured there would be high health care costs, drawn from common funds (= great size of consumption of common resources). Last assume that I am well aware of all of this (= high insight). If nevertheless I decide in this situation to go rock climbing, this would amount to a clear case of imprudent health behaviour according to our suggested definition. This claim is based upon the assumption that the proposed desiderata function to differentiate, in an ethically relevant way, my decision to go rock climbing from, let us say, your decision to go for a walk in the park or watch a football game.

The stress on risk and size of consumption of communal resources in the formula above is based on the commitment in LEHC to protect the prudent against unfair opportunity cost. Conversely, the stress on insight and avoidability is based on the weight placed on choice as voluntary action in LEHC. These two axes of ethical value must be combined to create imprudence, in a sense that is coherent with the ethos of LEHC. Thus, I may be excused for a seemingly imprudent act that I carry out with high insight and easy avoidability, if the resultant risk and/or size of consumption of communal resources is very small. Conversely, low insight and/or avoidability may excuse what would, in terms of risk and size only, have been an imprudent act.

The formula above could be amended to include, as a further excusing factor, *beneficence*. If so, an action which would on every other parameter count as imprudent would be excused if it were carried out for the common good (that is, if the level of beneficence was high). This would make the model better equipped to handle the “fire fighter problem” (Anderson 1999). At the same time such an inclusion would bring back the very moral arbitrariness that setting up a stringent definition of imprudence seeks to avoid. Therefore, we caution against this. However, as this has been a matter of some importance in the previous literature, we will return to it in greater detail in the “Threshold setting” section below.

Notably, the formula above provides *no cut-off levels* for acceptable risk, size, insight or avoidability. The problems involved with threshold setting in LEHC will be further explored below. For now we merely wish to point out that the issue of threshold setting is different from the question of moral arbitrariness (although lack of appropriate threshold setting may result in moral arbitrariness). The latter deals specifically with the possibility, in theory, to differentiate the imprudent from the prudent in LEHC theory. As seen, we believe that this particular problem can be solved^7^. However, LEHC faces other difficulties that remain even if the above framework is accepted. We will now turn to one of these.

## The “Ex Post” policy approach

With a workable definition of imprudence in place, LEHC may move on to define its scope of implementation. One crucial decision is whether to target impudent individuals at the time of consumption/risk-taking or at the time of claim to health care. These may be called the *ex ante* and the *ex post* policy approaches, respectively, and are commonly referred to in the previous literature on LEHC (see for instance Cappelen and Norheim 2005). These approaches may of course be mixed to form hybrid versions, but collectively they seem to exhaust the possible modes of application for LEHC. Unfortunately for LEHC, we find both the *ex post* and the *ex ante* approach unsuitable as expressions of the LEHC ethos. We will begin by describing the *ex post* approach and why it is problematic from the LEHC perspective.

In the *ex post* policy approach to LEHC, the issue of possible imprudence in health arises when a patient presents with a claim to health care. In that situation it becomes incumbent upon health care to establish whether the patient has been imprudent, in the relevant sense, and make health care prioritizations accordingly. Unfortunately for LEHC, the *ex post* policy approach predictably runs into epistemic difficulties (Sharkey and Gillam 2010). This is because the ethos of LEHC hinges on a causal link (established or presumed) between the imprudent action and the negative outcome. Recall from our suggested definition of imprudence that imprudence arises due to a morally unacceptable consumption of common resources. I am imprudent if I risk the consumption of communal resources by behaving in a way that may cause me to claim health (and conditions of scarcity apply). The crux is that in medicine, the necessary causal link is hard to establish. Several reasons make this so.

First, it may be hard to ascertain whether I did at all undertake the relevant action (especially if I have an incentive to be dishonest about this—see for instance Hansson 2018). Moreover, it may be equally hard to ascertain whether I met the relevant threshold levels of insight and/or avoidability. Last, many disease processes operate by way of joint causation. That is, although it is easy to establish that smoking *contributes* to lung cancer at the group level (along with other parameters such as lung tissue resilience, genetic factors, family history, and exposure to asbestos and air pollutants, see for instance Cruz et al. 2011), it may be difficult to establish a sufficiently strong causal link in *my* individual case. Furthermore, several authors have argued that establishing this link may prove both expensive and damaging to the doctor-patient relationship (Ho 2008).

What is pointed out here constitutes a problem for LEHC as establishing this link is vital to the justification of LEHC. Recall that LEHC is all about the individual patient shouldering the consequences of *her own* imprudent actions (as opposed to somebody else’s actions). It does not do, as justification, to say that I should shoulder consequences that *may be* the results of my actions. This, obviously, implies that I *may be* shouldering the consequences of other peoples’ actions, which is precisely the kind of sharing of opportunity cost that LEHC wishes to avoid. Therefore, the plausibility of the *ex post* policy approach hinges on the possibility of solving all epistemic difficulties involved. How difficult, expensive and/or damaging to the doctor-patient relationship this would be is an empirical question, and we encourage proponents of LEHC to present empirical studies where these issues are investigated. Until we have a firm answer to this, there is reason to be sceptical.

However, even if the epistemic difficulties are solved, the *ex post* approach harbours a more fundamental problem. As has been noted above, the *ex post* policy approach starts, as it were, in the clinic. The health care official interviews for instance a group of lung cancer patients to establish who among them have behaved imprudently (say, by reckless smoking). However, as it seems, it is a matter of brute luck whether *my* smoking results in lung cancer or not (Bognar 2019; Lippert-Rasmussen 2001). To be sure, other individuals behaved just as imprudently (that is, smoked just as much as I did) yet did not develop lung cancer. From my point of view, then, it will appear unjust (even doubly unjust) that my smoking first led to lung cancer *and* then to down-prioritizing for treatment, rather than to no disease at all. To be noted, this injustice is precisely of the kind that luck egalitarianism wishes to counteract, as there is no discernible morally relevant difference in imprudence between the smoker who developed lung cancer and the smoker who did not. To base priority decisions on the brute luck fact of whether the behaviour led to disease or not is rather like adding insult to burden, and it goes against the ethos of LEHC. This, we believe, provides a strong prima facie reason for LEHC to steer clear of the *ex post* policy approach.

## The “Ex Ante” policy approach

It may be more tempting, then, to try to operationalize LEHC via the *ex ante* approach. The most discussed example is *ex ante taxation* (Bærøe and Cappelen 2015; Le Grand 1991; Cappelen and Norheim 2005)^8^. In *ex ante* taxation, focus is shifted from *patients* to *risk takers—*or more to the point: it is shifted to those who *purchase products associated with risk taking*. Thus, so called “sin taxes” may be levied on the purchase of goods and actions that may give rise to (future) increased consumption of health care resources (Fonseca 2019). It should be noted that under the reading that sticks closest to the ethos of LEHC, the goal of such *ex ante* taxation is not primarily to dissuade individuals from engaging in certain (risky) practises, but rather to shore up resources that may finance the expected increase in consumption of health care resources. This way, the prudent can be protected from shouldering the opportunity costs that result from the actions of the imprudent, which is of course the point of the *luck* part of luck egalitarianism.

*Ex ante* taxation would seem to evade the problem of epistemic difficulty, as the tax is levied only from those that evidently perform the relevant purchases. However, things are not as straightforward as they seem. First, it is hard to tax non-behaviour and therefore *ex ante* taxation will predictably miss a large group of imprudent patients, for instance, those who do not pay for safety precautions (such as adequate gear for rock-climbing) or fail to do any physical exercise. When it comes to implementing *ex ante* policy, it is unjust from the point of view of LE to those people whose imprudent behaviour is taxable that they should pay such taxes, whereas other people with non-taxable imprudent behaviours are let off the hook. This injustice is a matter of brute luck, and thus presents the same problem as the one discussed regarding the *ex post* approach above^9^.

Second, *ex ante* taxation brings in more epistemic difficulties. This has to do with the fact that *ex ante* taxation cuts the abovementioned conceptual link between (imprudent) action and (negative) outcome. To see why, let us turn back to our proposed definition of imprudence. If a public health official buys a packet of cigarettes with the intention of using them as props in an anti-smoking campaign, there is no imprudence in the sense relevant to LEHC (as it does not lead to an increase in consumption of common resources). Therefore, it cannot be justified, on the basis of LEHC, to tax the health official’s purchase (although it may of course be justified in other ways). The former rock climber who buys an entrance ticket to the rock climbing park merely to evoke fond memories, or the minimalist cigar lover who smokes precisely one cigar each year are further cases in point. As there is no risk of increased consumption of communal resources in these cases^10^, it simply does not make sense to invoke risk as justification for taxing these people’s purchases. There may, of course, be other reasons to tax such purchases, but from a LEHC point of view such purchases are *prudent,* not imprudent. Therefore, LEHC should object rather than endorse taxation in cases such as these. This leaves LEHC with two options: drop *ex ante* taxation or face the epistemic difficulties all over. For if we want to be sure to tax only the smoker who smokes the right amount of cigars, we will have to establish a way of knowing how much he/she has smoked and will smoke—at the moment of purchase. This epistemic difficulty may prove even more difficult than in the *ex post* approach.

Yet another difficulty with the *ex ante* policy approach also stems from the severing of the link between action and outcome. If, for argument’s sake, we accept that the imprudent should pay extra taxes on their imprudent consumption, we must consider which extra consumption of communal resources these taxes should offset. To be true to the notion of opportunity cost, the net should be cast wide here. Imagine I am a smoking parent. By smoking I expose my children to tobacco smoke, thereby increasing their risk of asthma. This creates an increased demand for paediatric asthma care. It seems reasonable, from a LEHC point of view, that I should offset this increased demand by paying extra taxes on my cigarette consumption. Such tax policy will, however, affect equally my smoking friend who has no children. He, too, will have to co-finance paediatric asthma care whenever he buys a packet of cigarettes. Although other ethical standpoints may consider such an implication just, LEHC must perceive such an implication as plainly unjust. The problem is that *ex ante* taxation is not merely a way for me to shoulder the increased costs incurred by my imprudence, but partly also for me to shoulder the costs incurred by other people’s imprudence. This, of course, is exactly the kind of cost sharing that is perceived as unjust from a LEHC perspective.

To sum up and move on, we believe that the claim to justice in LEHC hinges upon a strong link—conceptually as well as epistemically—between the (imprudent) action and the (negative) outcome. As the *ex ante* policy approach severs the action-outcome link, it appears ill suited as expressions of LEHC. However, as explained above, the *ex post* policy approach is plagued by another set of difficulties involving epistemic difficulty and the paradox of targeting only a subset of the imprudent patients. In this sense, LEHC is permanently stuck between a rock and a hard place.

## Setting policy thresholds and abandoning whom?

Elizabeth Anderson famously discusses “abandonment” in conjunction with LEHC (Anderson 1999). She mentions both “abandonment of negligent victims” and “abandonment of the prudent”. Seeing that the whole point of LEHC is to target the negligent or imprudent—whether they be “victims” (as in the *ex post* policy approach) or not (as in the *ex ante* policy approach)—we believe that this charge is no prime concern for LEHC. The problem of “abandonment of the prudent” however is of great matter. To “abandon the prudent” will mean different things in the *ex post* and *ex ante* policy approach, respectively, but in both cases, it amounts to a risk that the prudent shoulders the costs of the imprudent. This is of course the kind of cost sharing that LEHC is meant to avoid. Therefore, the main task for LEHC, when it comes to threshold setting, is to avoid “pampering the imprudent” and “abandoning the prudent”. LEHC thus needs to provide policy rules which function to target all or nearly all (truly) imprudent, while making sure that non-imprudent individuals are not targeted. Some of the difficulties with this have been mentioned previously and concern conceptual matters such as the definitions of “high” in the “high risk” desiderata etc. Now we proceed to discuss the challenge of threshold setting from the outcome perspective and look at who will be targeted by LEHC policy depending on how the thresholds are set.

Let us first imagine a scenario where the thresholds for risk, insight, etc. are set *high*. This would allow down-prioritization of imprudent patients only in such cases where they had, for instance, truly high insight regarding the risks of their actions. The upside here is that there would be no complaints about “abandoning negligent victims”, and the policy would be coherent with the egalitarian aspect of LE. The downside is that the health care policy recommended in this scenario would be barely discernible from other, non-LEHC oriented health care policy. If one cares about the ethos of LEHC in the first place, it seems odd to advocate a solution where the ethos is made almost irrelevant to policy. Another problem is that such policy would treat some imprudent patients equally to the prudent patients. This, in turn, risks entailing precisely the kind of opportunity costs that LEHC wishes to avoid.

It makes sense, therefore, for LEHC to set the relevant thresholds *low*. For instance, intermediate risk of an intermediate size of consumption, intermediate insight and intermediate avoidability may suffice to classify an action as imprudent. This would have the advantage of striking those who are, presumably, the straightforward targets for LEHC policy: smokers with lung disease, alcoholics with liver disease, and overweight patients with type 2-diabetes. A low thresholds version of LEHC will thus avoid “pampering the imprudent”, who might go under the radar in the high thresholds version described above. At the same time and by the very same token some other and perhaps less self-evident targets will also be struck. Cases in point may be the myocardial infarction patient who is found to have stressed at work (as this increases the risk of myocardial infarction (Li et al. 2016)), the malignant melanoma patient who has waited “too long” before showing her odd mole to her GP (as this increases the risk of metastatic spread), and the flu patient who has looked after his grandchildren (as this increases the risk of infections).

We believe that such consequences will be counterintuitive and off-putting to many lay people. This is the “abandonment of negligent victims” discussed by Anderson. As we have mentioned, we presume that many who champion the ethos of LEHC will not be so easily put off, as consequences such as these flow naturally from the ethos of LEHC. Those who champion LEHC may nevertheless find it difficult to explain to the lay people why the weight placed on the LEHC ethos should be so large as to outweigh what is seen as strongly negative consequences. This provides, if nothing else, a pragmatic reason against LEHC.

More interesting is that consequences such as the ones described above may be perceived as “abandoning the prudent”, even by some who favour the LEHC ethos. Shlomi Segall, a leading LEHC theorist, provides a case in point (Segall 2009). Segall, a self-confessed value pluralist, recommends that some imprudent-like behaviour may be excused (as a matter of policy) if it results “from factors that it would be unreasonable to expect the agent to avoid” (Segall 2009). As can be seen, this is a substantial departure from our proposed definition of imprudence (where we used avoidability in a descriptive rather than in a normative sense). We find this suggestion problematic for several reasons.

The first is that referring to “what would be unreasonable” invites precisely those charges of moral arbitrariness that our definition of imprudence sought to avoid (Bognar 2019). That health care officials should define what is “unreasonable to avoid” seems implausibly paternalistic. However, even to define “unreasonable to avoid” in terms of what is considered normal and acceptable within a certain society at a certain point of time is morally arbitrary, as it presupposes that the current conventions are morally correct. The second problem is that the normative usage of the word avoidability implicit in Segall’s “unreasonable to avoid” clause sits uneasily next to our proposed more neutral definition of imprudence. This may of course point to a weakness in our definition. However, we believe it does not, but rather shows why the “unreasonable to avoid” clause invites relevant charges of moralism and/or moral arbitrariness. There are two ways out here. Either LEHC must explain the moral weight of “reasonableness” in a way which makes it a part of the overarching LEHC project and not merely an ad hoc solution to a failure of the theory (Fonseca 2019). Otherwise LEHC must go value pluralist and accept “reasonableness” as an independent normative concept without ties to the LEHC ethos. However, this solution risks reducing the ethos of LEHC to relative irrelevance, in a similar fashion as described above. If many of the natural consequences of LEHC are practically barred, then it becomes difficult to see why the ethos of LEHC should be invited into policy at all.

## Conclusion

In this article we have attempted to discuss some problems that ensue when the ethos of luck egalitarianism is set to practise in health care. In so doing we have, for the sake of argument, accepted the luck egalitarian ethos at face value. We argue, however, that even those who support this ethos will balk at the difficulties involved in the real-world implementation of LEHC. Although the charge of moral arbitrariness may well be countered, at least to a larger degree than has been recognized by LE’s critics, we think that neither the *ex ante* nor the *ex post* policy approach accurately captures the LEHC ethos—yet we see no third alternative. Furthermore, setting a stringent LEHC threshold will be difficult and risk “pampering the imprudent”, “abandoning the prudent”, or worst, both of these. In the face of the threshold setting problems, it may be tempting for LEHC to resort to measures which circumscribe the policy impact of the theory gravely, or go value pluralist and combine LEHC with some other normative material. In either of these ways, the LEHC ethos will be reduced to second-hand normative importance.

We have made a serious attempt to operationalize the ethos of luck egalitarianism in health care in a just way. Due to tensions within the theory, and the complex nature of health care, our attempt has failed. Our conclusion is that luck egalitarianism cannot be consistently applied as a convincing and relevant normative principle in health care policy.

## Footnotes

1 It may be possible to apply luck egalitarianism to *health itself,* rather than health *care*. However, this article is solely focused on how luck egalitarianism may be applied to health care.

2 From here on, we will for reasons of text economy say instead that “LEHC would hold”.

3 The terms “imprudent” and “imprudence” are often used in the literature on LEHC to denote the person who behaves irresponsibly in health, and this behaviour, respectively. In this article, we will use “imprudent” and “imprudence” in this sense.

4 Although there are obvious differences between the possible health costs to prudent patients and the possible economic costs to prudent tax payers (or insurance premium payers), we consider the ethical implications of these two “costs” to be similar enough for our present purposes. Therefore, we will not discern between the two types of “cost” in the following analysis.

5 For more on desertism, and how this theory differs from luck egalitarianism, see Brouwer and Mulligan (2019). Here it may suffice to point out that for LEHC, unlike for desertism, imprudence is a function of opportunity cost. The conceptual link between generating opportunity cost and being imprudent is employed in our proposed definition of imprudence, see below.

6 We assume here that willful ignorance does not count as low insight and is therefore no protection against charges of imprudence. In a sense, then, our suggestion implies that individuals have some sort of responsibility to gain relevant insights. This is not in itself a controversial stance, we believe, but it does open up a further set of threshold issues.

7 At least to a larger extent than suggested by some of the critics of LEHC (Wilkinson 1999; Wikler 2002). In the end, any attempt to adopt LE into health care policy will result in different kinds of arbitrariness, or so we will argue below. But the problems with LEHC are not the commonly assumed ones: we have here suggested how these can be tackled by LEHC.

8 Another possible *ex ante* policy approach is differential insurance premiums. However, the same kind of fundamental challenges arise regardless of whether the *ex ante* policy is applied to taxation or insurance premiums.

9 As soon as the policy is firmly set in place this, of course, ceases to be a matter of brute luck. As soon as the policy is set up, so that it is evident which imprudent behavior is taxed and which is not, my choosing to engage in the one or the other is a matter of option luck (provided one is not held responsible for one’s taste, see for instance Dworkin 1981). However, this does not help LEHC much. True, the *ex ante* approach converts some previously imprudent actions into prudent actions (as they are taxed, and do not lead to opportunity cost). Yet the untaxable imprudent actions remain untouched by the *ex ante* approach, and so there is still cost shifting from the imprudent to the prudent.

10 A recent article takes this fact as a departure for a discussion about “diachronicity” in responsibility-sensitive policy (Brown and Savulescu 2019). The discussion does not alter what is said here, however.
